# Longitudinal associations between perceived benefits and costs of internet gaming and internet gaming disorder in adolescent gamers: A cross‑lagged structural equation model

**DOI:** 10.1371/journal.pone.0351550

**Published:** 2026-06-09

**Authors:** Xue Yang, Xin Wang, Miguel Ribeiro Ramos

**Affiliations:** 1 The Jockey Club School of Public Health and Primary Care, Faculty of Medicine, The Chinese University of Hong Kong, Hong Kong SAR, China; 2 Department of Social Policy, Sociology and Criminology, University of Birmingham‌‌, Birmingham, United Kingdom; Yale University, UNITED STATES OF AMERICA

## Abstract

This longitudinal panel study aims to investigate the relationships between short/long-term benefits and costs of internet gaming and internet gaming disorder (IGD) in adolescents. First-year high school students with a convenience sampling were recruited from four high schools in central China, and 1032 (56% boys) finished baseline and one-year follow-up surveys. The percentage of IGD was 14.8% at T1 and 13.9% at T2. Cross-lagged structural equation modeling showed that higher levels of IGD symptoms predicted more perceived short- and long-term costs of gaming, while perceptions of short- and long-term costs or benefits did not significantly predict IGD symptoms. These findings suggest experiencing actual negative consequences of internet gaming could enhance perceived costs. Prospective associations between cognitive perceptions and IGD are highlighted and the implications for interventions are discussed.

## Introduction

Internet gaming is a double-edged sword with both benefits and costs. Play is not merely about fun but also has social and developmental functions for youth [1]. In this digital age, playing internet games has become one of the main activities and means for entertainment, peer interactions and connectivity, social competence, positive self-feelings, boredom relief, and relaxation in youth [2]. Research has contended that playing video games may have cognitive benefits (e.g., enhanced spatial skill, neural processing and efficiency, problem-solving skills, and creativity), motivational benefits (e.g., optimistic beliefs about their intelligence, abilities, and failure), emotional benefits (e.g., positive mood, relaxation, and warding off anxiety), and social benefits (e.g., prosocial skills and civic engagement) [3]. However, internet gaming may have costs and negative consequences, such as cybersecurity risks, financial costs, sedentary lifestyle, weakened real-world social skills and exposure to toxic online environments [4,5]. Particularly, excessive internet gaming has been widely found to relate with sleep problems, emotional distress, aggressive behaviors, academic stress, and interpersonal problems [6–8]. Internet gaming disorder (IGD) has been included as a condition that needs to be further studied in the Fifth Edition of the Diagnostic and Statistical Manual of Mental Disorders (DSM-5), which is characterized by symptoms of preoccupation, withdrawal, tolerance, loss of control, loss of interest, continued overuse, deceiving, escape of negative feelings, and functional impairment. In particular, loss of interest refers to loss of interest in non-gaming activities, while continued overuse refers to continued overuse of Internet games despite knowledge of psychological and social concerns. Gaming disorder is also defined as a mental disorder in the 11th Revision of the International Classification of Disease (ICD-11) by the World Health Organization. Adolescents, especially in Asia, are at higher risk of IGD than adults. According to a meta-analysis of 16 studies, the pooled prevalence of IGD among adolescents was 4.6%; male adolescents reported a higher prevalence rate (6.8%) than female adolescents (1.3%), and Asian samples reported higher prevalence than other regions [9]. Another meta-analysis of 155 reports in 33 countries reported the pooled prevalence of IGD of 8.8% among adolescents with higher prevalence reported in Chinese populations [10].

### The roles of perceived benefits and costs in IGD‌‌

Perceptions and beliefs about the benefits and costs of internet gaming may affect one’s decision on internet gaming behaviors and the risk of IGD. Specifically, perceiving high benefits and low costs of internet gaming may motivate individuals to rely on internet games. Social cognitive theory (SCT) emphasizes the processes involved in creating expectancies of others’ behaviors and how these expectancies guide one’s own actions. Accordingly, individuals may develop positive expectations about gaming through observing others’ experiences and rewards, such as peers’ enjoyment, achievements in games, or recognition within online gaming communities. Such observational learning may reinforce the perceived benefits of gaming and increase the likelihood of engaging in gaming behaviors. Conversely, perceiving negative consequences, such as punishment, associated with gaming may reduce individuals’ likelihood of engaging in those behaviors [11]. The theory of planned behavior (TPB) also suggests that positive attitudes toward a behavior (e.g., positive behavioral beliefs and perceived behavioral outcomes) would shape an individual’s behavioral intentions [12]. Consistently, the health belief model (HBM) highlights that if an individual believes that a particular action will benefit him or her, then he or she is likely to engage in that behavior [13]. For example, if people believe that internet gaming can benefit them by reducing worries, they are more likely to rely on internet gaming for stress coping. In the present study, we operationalize these theoretical constructs by measuring four specific outcome expectancies: immediate positive (short-term benefit), immediate negative (short-term cost), delayed positive (long-term benefit), and delayed negative (long-term cost). This differentiation allows us to test whether time preference—the relative weighting of immediate versus delayed outcomes—plays a role in IGD, as suggested by decision theory and economic models of addiction [14]. A few cross-sectional studies have reported a positive correlation between positive beliefs of internet gaming (e.g., positive outcome expectancy of internet gaming) and IGD [15–18]. A study on metacognitions about internet games found that positive metacognitions about internet gaming (e.g., emotional benefits: “internet gaming stops me from worrying”) were not associated with IGD, while negative metacognitions about the uncontrollability of internet gaming (e.g., “I have no control over how much time I play”) and the dangers of internet gaming (e.g., “Thoughts about online gaming are becoming an obsession”) were significantly positively associated with IGD [19,20]. However, we did not find any longitudinal studies testing related perceptions and IGD. It remains unknown whether such perceptions would predict IGD symptoms or vice versa.

### The roles of IGD in affecting perceived benefits and costs of internet gaming

It is possible that experiencing IGD symptoms may reduce the perceived benefits and enhance the perceived costs of internet gaming. As a mental disorder and addictive problem, patients with IGD may exhibit preoccupation with internet gaming even during other activities, an inability to abstain from internet gaming, dissatisfaction even after excessive gaming, loss of self-control and interest in other daily-life activities, and may suffer from emotional distress, health problems, academic difficulties, and interpersonal conflicts [21]. Experiencing these symptoms and negative consequences can be painful and may have direct impact on reducing the perceived benefits of internet gaming and enhancing the negative perceptions of internet gaming. However, there were no studies examining whether increased IGD symptoms would change how adolescents who game think about internet gaming regarding its benefits and costs.

### Time preference

Time preference (i.e., the relative valuation of short-term versus long-term benefits and costs) has been found to be related to addictive behaviors (e.g., smoking, drinking, opioid use disorder, and problem gambling) [14]. According to the decision theory and economic theory [22,23], tradeoffs are resolved by the value placed on delayed costs relative to more immediate benefits. Thus, a potential explanation for why individuals engage in internet gaming is that they steeply discount long-term costs (e.g., health problems) due to delay and uncertainty, while attributing more value to immediate benefits (e.g., escaping from real-life stress, relaxing). Review studies showed strong evidence for the correlations between time preference measures and addictive behaviors, including substance (e.g., smoking) and non-substance behaviors (e.g., gambling) [14,24]. However, to our knowledge, there were no longitudinal or experimental studies testing the roles of short-term versus long-term benefits and costs in IGD.

### The present study

The present study investigated the potential reciprocal relationships between the levels of perceptions of benefits and costs of internet gaming and IGD symptoms in a one-year longitudinal survey among Chinese adolescent who game. Furthermore, we aimed to differentiate the roles of the perceptions regarding short-term versus long-term benefits and costs in these relationships. It is hypothesized that perceiving high benefits (especially short-term benefits) or low costs (especially long-term costs) of internet gaming would increase IGD symptoms; in turn, IGD symptoms would reduce perceived benefits and enhance perceived costs of internet gaming. The study also examined the prevalence and persistent and remission rates of IGD and the changes in the levels of perceived short-term versus long-term benefits and costs over time in our sample of Chinese adolescent who game.

## Method

### Participants and procedure

The longitudinal panel data with convenience sampling were collected from four high schools in central China. The research team initially contacted the school principals and headteachers to explain the study objectives and obtain institutional approval. The inclusion criteria for participation were: 1) first-year high school students; 2) willingness to finish the surveys at baseline and follow-up, 3) providing both parental and students’ informed consent, 4) Chinese speakers, and 5) playing internet games in the past year. The exclusion criteria included being participants with learning disabilities or those who provided random answers to the survey (i.e., selecting the identical response option (e.g., all ‘1’)). The baseline survey was conducted in Oct 2018, during the participants’ first year of high school (T1), while the one-year follow-up survey (T2) was conducted in Oct 2019 during their second year of high school. Participants completed the 15-minute surveys on paper in a classroom setting and with the assistance of research assistants. They were guaranteed that participation was voluntary, that there were no penalties for those who did not want to take part, and that it was confidential and anonymous. A code for each participant was generated for data matching. No incentive was given to the participants. The study was in accordance with the Strengthening the Reporting of Observational Studies in Epidemiology (STROBE) reporting guideline for cohort studies. The study was approved by the Survey and Behavioral Research Ethics Committee of the corresponding author’s affiliation (Ref# 055−18). Written informed consent was obtained from both participants and their parents.

At T1, 1,297 students were approached but 51 students refused to participate; 1,246 students completed T1 survey but 7 of them gave random answers. At T2, 1,205 of the students completed the follow-up survey but 5 of them gave random answers; thus, 1,200 students completed both surveys. After excluding the data of individuals who did not game, 1032 participants were enrolled into analysis of this study. Half of the participants (56.0%) were male. The mean age of the participants was 15.60 (SD = .61). Most participants were born in the urban area (84.8%) and lived with both parents (85.1%). In terms of education, over 40.0% of the participants’ parents had university education. The family socio-economic status of the sample was 33.4% ordinary or below, 51.6% good, 8.7% very good and 6.2% refusing to answer (Table 1).

**Table 1:** Background characteristics of the participants (N = 1032).

	Frequency (%)
Gender	
Male	578 (56.0)
Female	454 (44.0)
Born in	
Urban area	875 (84.8)
Rural area	157 (15.2)
Living with	
Father	34 (3.3)
Mother	80 (7.8)
Both	878 (85.1)
Neither	40 (3.9)
Family socio-economic status	
Ordinary or below	345 (33.4)
Good	533 (51.6)
Very good	90 (8.7)
Refuse to answer/unknown	64 (6.2)
Father’s education level	
Junior high school or below	128 (12.4)
Senior high school to college	399 (38.7)
Undergraduate or above	486 (47.1)
Refuse to answer/unknown	19 (1.8)
Mother’s education level	
Junior high school or below	154 (14.9)
Senior high school to college	405 (39.2)
Undergraduate or above	451 (43.7)
Refuse to answer/unknown	22 (2.1)

### Measures

Perceived short-term versus long-term benefits and costs of internet gaming were measured by four adapted questions used in previous studies on other addictive behaviors, such as smoking and sugar consumption [14,25] (“To what extent do you believe that internet gaming has a *positive* impact *at present* on you?”, “To what extent do you believe that internet gaming has a *negative* impact *at present* on you?”, “To what extent do you believe that internet gaming has a *positive* impact *in the future* on you?” and “To what extent do you believe that internet gaming has a *negative* impact *in the future* on you?”). The items are rated on a 10-point numeric rating scale, ranging from 1 = none to 10 = extremely strong.

IGD symptoms were measured by the Chinese version of the 9-item DSM-5 IGD Symptoms checklist [26,27]. It is a short, user-friendly, self-report measure assessing IGD symptoms in the past 12 months. Response options include no (0) and yes [1]. Higher sum scores suggest higher levels of IGD symptoms, with possible scores in this measure ranging from 0 to 9. A score of 5 is taken as the cutoff point for defining probable IGD based on DSM-5 criteria [27]. The scale reliability was good in the present study (Cronbach’s alpha was  .76 at T1 and  .83 at T2).

### Statistical analysis

Univariate linear regression models were used to assess the association between background factors and IGD symptoms at T1. Pearson’s correlation coefficients were used for the correlations among the perceived short-term benefits, short-term costs, long-term benefits, long-term costs, and IGD symptoms at T1 and T2.

Structural equation modelling (SEM) was conducted to test the cross-lagged effects of perceived benefits, perceived costs and IGD symptoms, adjusting for significant background factors. The cross-lagged analysis allowed us to test the directionality of our variables across the two measured time points [28]. The measurement model was tested using confirmatory factor analysis (CFA), which examined the goodness-of-fit of the pattern of observed indicators for the latent constructs in the proposed model. The hypothesized directionality of the associations among the constructs and the overall fit of the model were examined in the SEM analysis. As recommended by previous studies, χ2/df ≤ 3, the Comparative Fit Index (CFI)>.90, the Tucker-Lewis Index (TLI)>.90, the Root Means Square Error of Approximation (RMSEA)<.06 and the Standardized Root Mean Square Residual (SRMR)<.08 were used to assess the model fit [29,30]. To reduce model complexity and improve estimation stability in the longitudinal SEM, item parcels of IGD symptoms were created following a procedure recommended by Russell, Kahn [31]. The nine items of the scale were first factor-analyzed. Items were ranked in order based on the size of their factor loadings and subsequently assigned to one of three item parcels. Specifically, items ranked 1, 5, and 9 were assigned to the first parcel, items ranked 2, 6, and 8 to the second parcel, and items ranked 3, 4, and 7 to the third parcel. This procedure results in item parcels reflecting the measured construct to an equal degree [31]. Short-term and long-term benefits and costs, as well as IGD parcels, were treated as continuous variables in the model.

The data significantly deviated from multivariate normality (skewness values ranging from −.10 to 7.18 and kurtosis values ranging from −2.00 to 49.56, with the highest skewness and kurtosis values for father’s education level; Mardia’s multivariate kurtosis coefficients = 126.62). To address the non-normality of our data, the analysis utilized maximum likelihood estimation with robust standard errors (MLR) to ensure robust parameter estimates. Statistical significance was set at the  .05 level. SPSS 27.0 Statistics for Windows and Mplus 7.4 were used for all statistical analyses. In terms of missing data,  .03%−.04% missing data for short-term/long-term benefits and costs at T1 were handled using series mean imputation. For background factors, missing values (1.8%−6.2%) were regrouped in one category (e.g., Family socio-economic status: 1 = Ordinary or below, 2 = Good, 3 = Very good, 4 = Refuse to answer/unknown). Regarding sample size adequacy, it is suggested that 10:1 ratio of sample size to the number of free parameters [32]. In our SEM model, there were 87 free parameters. Our sample size (N = 1032) was enough to test the hypothesized model.

## Results

### Preliminary analyses

The paired t-test revealed that short-term and long-term benefits at T2 were significantly higher than those at T1 [Short-term benefits: mean of change = .68, Standard Deviation (SD)=2.64, *t*-Statistic = 8.27, *p* < .001; long-term benefits: mean of change = .53, SD = 2.64, t-Statistic = 6.42, *p* < .001]. Accordingly, the short-term and long-term costs at T2 were significantly lower than those at T1 [Short-term costs: mean of change = −.38, SD = 2.80, *t*-Sta*t*istic = −4.34, *p* < .001; long-term costs: mean of change = −.62, SD = 2.92, *t*-Statis*t*ic = −6.76, *p* < .001].

The percentage of IGD was 14.8% at T1 and 13.9% at T2. In terms of change and stability of IGD over 1 year, the percentages of persistent IGD (IGD at T1 and T2), incident IGD (non-IGD at T1 but IGD at T2), remission from IGD (IGD at T1 but non-IGD at T2) and persistent non-IGD (non-IGD at T1 and T2) were 5.7%, 5.4%, 8.6% and 80.2%, respectively. However, no significant difference in the mean scores of IGD symptoms between T1 and T2 was found.

Results of univariate linear regression showed that adolescents who were female (*β* = −.18, *p* < .001) and had good family socio-economic status (*β* = −.10, *p* < .01) reported lower levels of IGD symptoms. Furthermore, the higher father’s educational level (undergraduate or above: *β* = −.10, *p* = .049) and mother’s educational level (senior high school to college: *β* = −.19, *p* < .001; undergraduate or above: *β* = −.16, *p* < .001) were associated with lower levels of IGD symptoms at T1 (Table 2).

**Table 2:** Associations between background variables and IGD symptoms at T2 by univariate linear regression models (N = 1032).

	*β*	B	SE	95%CI
Age	−.01	−.03	.10	(−.23, .17)
Sex (male ref. vs. female)	−.18	**−.71**	**.12**	(−.95, −.48)
Born in urban area (yes ref. vs. no)	.01	.04	.17	(−.29, .38)
Living with both parents (no ref. vs. yes)	−.03	−.17	.17	(−.51, .17)
Family socio-economic status (ref. ordinary or below)				
Good	−.10	**−.39**	**.14**	(−.66, −.12)
Very good	−.03	−.24	.23	(−.70, .22)
Refuse to answer/unknown	.04	.31	.27	(−.22, .83)
Father’s education level (ref. junior high school or below)				
Senior high school to college	−.07	−.27	.20	(−.66, .13)
Undergraduate or above	−.10	**−.39**	**.20**	(−.77, −.001)
Refuse to answer/unknown	−.08	**−1.12**	**.49**	(−2.08, −.17)
Mother’s education level (ref. junior high school or below)				
Senior high school to college	−.19	**−.77**	**.19**	(−1.14, −.41)
Undergraduate or above	−.16	**−.65**	**.18**	(−1.01, −.29)
Refuse to answer/unknown	−.06	−.85	.45	(−1.73, .03)

β = Standardized coefficients; B = Unstandardized coefficients; SE = Standard error; ref. = Reference group; 95%CI = 95% confidence intervals. Statistically significant background variables were highlighted in bold.

Table 3 presents the descriptive statistics (mean and SD) and the Pearson correlations for the key variables in this study. Results showed that perceived short-term and long-term benefits at T1 were significantly and positively correlated with IGD symptoms at T2. While, perceived short-term and long-term costs at T1 were not correlated with IGD symptoms at T2. Perceived benefits and perceived costs were significantly and inversely correlated with each other at T1 and T2.

**Table 3:** Correlations between perceived short-term benefits, short-term costs, long-term benefits, long-term costs, and IGD symptoms at T1 and T2 (N = 1032).

		Range	M(SD)	Perceived short-term benefits		Perceived short-term costs		Perceived long-term benefits		Perceived long-term costs		IGD scores	
				T1	T2	T1	T2	T1	T2	T1	T2	T1	T2
Perceived short-term benefits	T1	1-10	4.44 ± 2.34	--									
	T2	1-10	5.12 ± 2.17	**.32** ^***^	**--**								
Perceived short-term costs	T1	1-10	5.04 ± 2.52	**−.38** ^***^	**−.22** ^***^	**--**							
	T2	1-10	4.66 ± 2.17	**−.19** ^***^	**−.31** ^***^	**.29** ^***^	**--**						
Perceived long-term benefits	T1	1-10	4.09 ± 2.32	**.76** ^***^	**.27** ^***^	**−.33** ^***^	**−.19** ^***^	**--**					
	T2	1-10	4.62 ± 2.21	**.30** ^***^	**.76** ^***^	**−.18** ^***^	**−.29** ^***^	**.32** ^***^	**--**				
Perceived long-term costs	T1	1-10	5.76 ± 2.58	**−.33** ^***^	**−.16** ^***^	**.76** ^***^	**.26** ^***^	**−.42** ^***^	**−.19** ^***^	**--**			
	T2	1-10	5.15 ± 2.37	**−.20** ^***^	**−.31** ^***^	**.30** ^***^	**.77** ^***^	**−.22** ^***^	**−.39** ^***^	**.31** ^***^	**--**		
IGD symptoms	T1	0-9	2.06 ± 2.12	**.36** ^***^	**.17** ^***^	.001	**.07** ^*^	**.28** ^***^	**.13** ^***^	.02	.05	**--**	
	T2	0-9	1.96 ± 1.98	**.18** ^***^	**.30** ^***^	−.01	**.07** ^*^	**.14** ^***^	**.21** ^***^	.03	.05	**.46** ^***^	--

M = Mean; SD = Standard deviation; IGD = Internet gaming disorder. ^*^p < .05; ^**^p < .01; ^***^p < .001.

### Structural equation modelling

**Fig 1:**
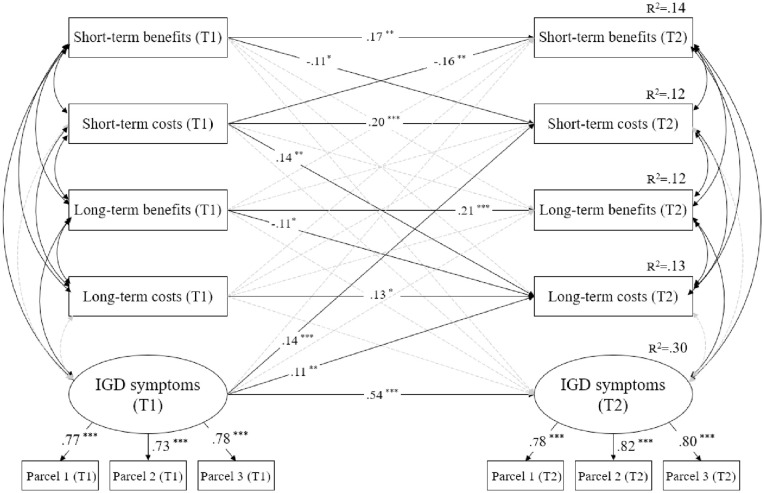
SEM of the cross-lagged effects of perceived short-, long-term benefits, costs, and IGD symptoms. *Note.* IGD = Internet gaming disorder. The standardized path coefficients were‌‌ shown in the Fig 1. The background variables (i.e., gender, family income and parental education levels) were adjusted for. * *p* < .05, ** *p* < .01, *** *p* < .001.

The measurement model yielded a good fit: Satorra-Bentler χ2/df = 1.76, CFI = .99, TLI = .98, RMSEA = .03 (95%CI = .02−.03), SRMR = .01. All the parcel indicators were significantly loaded on the latent variables, with standardized factor loadings ranging from  .72 to  .82 (all *p* < .001). With a Satorra-Bentler χ2/df = 1.93, CFI = .97, TLI = .96, RMSEA = .03 (95%CI = .02−.04), SRMR = .03, the tested SEM model showed a good fit. As shown in Fig 1 and Table 4, IGD symptoms at T1 were positively associated with perceived short- and long-term costs at T2 (*β* = .14, 95%CI = .07,  .21; *β* = .11, 95%CI = .04,  .18), but did not affect perceived short- or long-term benefits at T2. However, perceived short- and long-term benefits and costs at T1 were not significantly associated with IGD symptoms at T2. Perceived short-term benefits at T1 showed a significant negative longitudinal relationship with the perceived short-term costs at T2 (*β* = −.11, 95%CI = −.21, −.01). Furthermore, perceived short-term costs at T1 was inversely associated with perceived short-term benefits at T2 (*β* = −.16, 95%CI = −.26, −.05) and positively associated with perceived long-term costs at T2 (*β* = .14, 95%CI = .04,  .24). Perceived long-term benefits at T1 had a negative association with perceived long-term costs at T2 (*β* = −.11, 95%CI = −.21, −.02).

**Table 4:** Unstandardized and standardized path parameter estimates, and factor loadings for the SEM model (N = 1032).

Path	B (SE)	*β* (95%CI)
Perceived short-term benefits (T1) → Perceived short-term costs (T2)	**−.11 (.05)** ^*^	−.11 (−.21, −.01)
Perceived short-term benefits (T1) → Perceived long-term benefits (T2)	.09 (.05)	.09 (−.004, .19)
Perceived short-term benefits (T1) → Perceived long-term costs (T2)	−.07 (.05)	−.07 (−.16, .03)
Perceived short-term benefits (T1) → IGD symptoms (T2)	−.02 (.02)	−.08 (−.18, .03)
Perceived short-term costs (T1) → Perceived short-term benefits (T2)	**−.13 (.05)** ^**^	−.16 (−.26, −.05)
Perceived short-term costs (T1) → Perceived long-term benefits (T2)	−.05 (.05)	−.06 (−.16, .04)
Perceived short-term costs (T1) → Perceived long-term costs (T2)	**.13 (.05)** ^**^	.14 (.04, .24)
Perceived short-term costs (T1) → IGD symptoms (T2)	−.02 (.01)	−.08 (−.18, .01)
Perceived long-term benefits (T1) → Perceived short-term benefits (T2)	.07 (.04)	.08 (−.02, .17)
Perceived long-term benefits (T1) → Perceived short-term costs (T2)	−.06 (.04)	−.07 (−.16, .02)
Perceived long-term benefits (T1) → Perceived long-term costs (T2)	**−.11 (.05)** ^*^	−.11 (−.21, −.02)
Perceived long-term benefits (T1) → IGD symptoms (T2)	.003 (.02)	.01 (−.09, .12)
Perceived long-term costs (T1) → Perceived short-term benefits (T2)	.03 (.04)	.03 (−.07, .14)
Perceived long-term costs (T1) → Perceived short-term costs (T2)	.03 (.04)	.04 (−.06, .14)
Perceived long-term costs (T1) → Perceived long-term benefits (T2)	−.03 (.05)	−.03 (−.13, .07)
Perceived long-term costs (T1) → IGD symptoms (T2)	.02 (.01)	.06 (−.04, .16)
IGD symptoms (T1) → Perceived short-term benefits (T2)	.21 (.12)	.07 (−.01, .14)
IGD symptoms (T1) → Perceived short-term costs (T2)	**.44 (.12)** ^***^	.14 (.07, .21)
IGD symptoms (T1) → Perceived long-term benefits (T2)	.09 (.12)	.03 (−.04, .10)
IGD symptoms (T1) → Perceived long-term costs (T2)	**.38 (.13)** ^**^	.11 (.04, .18)
		
Perceived short-term benefits (T1) → Perceived short-term benefits (T2)	**.16 (.05)** ^**^	.17 (.07, .27)
Perceived short-term costs (T1) → Perceived short-term costs (T2)	**.17 (.04)** ^***^	.20 (.10, .30)
Perceived long-term benefits (T1) → Perceived long-term benefits (T2)	**.20 (.05)** ^***^	.21 (.11, .30)
Perceived long-term costs (T1) → Perceived long-term costs (T2)	**.12 (.05)** ^*^	.13 (.03, .23)
IGD symptoms (T1) → IGD symptoms (T2)	**.52 (.05)** ^***^	.54 (.45, .63)
		
Perceived short-term benefits (T1) ↔ Perceived short-term costs (T1)	**−2.24 (.23)** ^***^	−.38 (−.45, −.31)
Perceived short-term benefits (T1) ↔ Perceived long-term benefits (T1)	**4.13 (.22)** ^***^	.76 (.73, .80)
Perceived short-term benefits (T1) ↔ Perceived long-term costs (T1)	**−1.97 (.23)** ^***^	−.33 (−.40, −.26)
Perceived short-term benefits (T1) ↔ IGD symptoms (T1)	**.64 (.06)** ^***^	.40 (.33, .47)
Perceived short-term costs (T1)) ↔ Perceived long-term benefits (T1)	**−1.91 (.22)** ^***^	−.33 (−.40, −.26)
Perceived short-term costs (T1)) ↔ Perceived long-term costs (T1)	**4.91 (.23)** ^***^	.76 (.71, .80)
Perceived short-term costs (T1) ↔ IGD symptoms (T1)	.004 (.07)	.002 (−.07, .08)
Perceived long-term benefits (T1) ↔ Perceived long-term costs (T1)	**−2.53 (.23)** ^***^	−.42 (−.49, −.36)
Perceived long-term benefits (T1) ↔ IGD symptoms (T1)	**.50 (.07)** ^***^	.31 (.24, .39)
Perceived long-term costs (T1) ↔ IGD symptoms (T1)	.03 (.07)	.02 (−.06, .09)
Perceived short-term benefits (T2) ↔ Perceived short-term costs (T2)	**−1.11 (.19)** ^***^	−.27 (−.36, −.18)
Perceived short-term benefits (T2) ↔ Perceived long-term benefits (T2)	**3.05 (.19)** ^***^	.74 (.69, .78)
Perceived short-term benefits (T2) ↔ Perceived long-term costs (T2)	**−1.18 (.19)** ^***^	−.27 (−.35, −.19)
Perceived short-term benefits (T2) ↔ IGD symptoms (T2)	**.31 (.05)** ^***^	.28 (.20, .36)
Perceived short-term costs (T2)) ↔ Perceived long-term benefits (T2)	**−1.01 (.18)** ^***^	−.24 (−.32, −.16)
Perceived short-term costs (T2)) ↔ Perceived long-term costs (T2)	**3.30 (.20)** ^***^	.74 (.69, .79)
Perceived short-term costs (T2) ↔ IGD symptoms (T2)	.04 (.05)	.03 (−.06, .12)
Perceived long-term benefits (T2) ↔ Perceived long-term costs (T2)	**−1.62 (.21)** ^***^	−.35 (−.44, −.27)
Perceived long-term benefits (T2) ↔ IGD symptoms (T2)	**.21 (.05)** ^***^	.19 (.11, .27)
Perceived long-term costs (T2) ↔ IGD symptoms (T2)	.02 (.05)	.02 (−.07, .10)
**Factor Loadings**		
IGD symptoms (T1) → Parcel score 1	1	.77 (.73, .81)
IGD symptoms (T1) → Parcel score 2	**.89 (.04)** ^***^	.73 (.68, .77)
IGD symptoms (T1) → Parcel score 3	**.86 (.04)** ^***^	.78 (.73, .82)
IGD symptoms (T2) → Parcel score 1	1	.78 (.74, .82)
IGD symptoms (T2) → Parcel score 2	**.94(.04)** ^***^	.82 (.78, .86)
IGD symptoms (T2) → Parcel score 3	**.99(.05)** ^***^	.80 (.76, .83)

SE = Standard error; 95%CI = 95% bias-corrected confidence intervals; IGD = Internet gaming disorder. ^*^ *p* < .05, ^**^ *p* < .01, ^***^ *p* < .001.

### Sensitivity analysis

We conducted sensitivity analyses for the SEM model using the summed IGD score and one dataset imputed by mice in R. The results were substantively consistent with those obtained from the parcel-based latent model, supporting the robustness of our findings (See S1 File).

## Discussion

This study represented the first longitudinal study examining the relationships between perceived short-term/long-term benefits and costs of internet gaming and IGD in adolescent who game. Our participants reported high prevalence of IGD at both time point (15% and 14%), consistent with previous studies in this age group [33]. Our findings also align with prior research indicating that male gender and lower family socioeconomic and educational status are associated with an increased risk of IGD [34–36]. This vulnerability may be attributable to factors such as peer influence, stress, and inadequate supervision. It is therefore crucial to implement tailored prevention and intervention strategies for these groups. Such efforts could be multi-tiered, including school-based universal prevention programs that enhance digital literacy and stress-coping skills to mitigate peer influence and stress and targeted family interventions that equip parents, particularly in low socioeconomic households, with skills to set healthy boundaries and provide alternative reinforcement.

Our findings reveal a nuanced temporal relationship between cognitive perceptions of gaming and IGD symptoms. While cross-sectional correlations replicate prior studies in showing concurrent associations between perceived benefits and IGD severity [15–18], the longitudinal cross-lagged analyses suggest an unidirectional relationship. Specifically, the lack of predictive effect from T1 cognitive perceptions (both benefits and costs) to T2 IGD symptoms indicates that, within the limits of a two-wave observational design, these perceptions may not be primary drivers in the development or escalation of IGD over time. Instead, the significant paths from T1 IGD symptoms to T2 perceived costs (both short- and long-term) are consistent with a symptom-driven cognitions model: once problematic gaming patterns are established, individuals become increasingly aware, or perhaps more willing to acknowledge, the negative consequences of their behavior. This pattern aligns with the insight process often observed in behavioral addictions, where the negative outcomes and perceived costs of the behavior become more salient as the disorder progresses [37–39]. Such a shift may reflect experience-dependent cognitive reappraisal: as adolescents accumulate real-world consequences from excessive gaming (e.g., academic decline, family conflict, sleep deprivation), their awareness of these costs increases, leading to recalibrated perceptions of gaming’s negative impact. This interpretation is consistent with the I-PACE model [40], which posits that repeated engagement in addictive behaviors leads to conditioned cognitive and emotional responses that become more accessible over time. This offers preliminary evidence that may refine cognitive-driver models that distorted cognitions drive addiction, and underscores the need for developmental frameworks in which cognitions follow rather than lead behavioral pathology. Future work should explore moderators of this symptom-to-cognition pathway, such as social feedback and functional impairment. Also, we did not find any time preference regarding the relationships between short-/long-term benefits/cost and IGD. It is different from the studies on other addictive behaviors, such as smoking [14]. Experimental studies may provide more insightful information of the roles of immediate gratification and punishment in IGD.

The study has several limitations. First, we recruited participants in central China using convenience sampling, which could be subjected to selection bias and limits the generalizability of our findings to other regions and cultures and clinical samples. Second, the use of self-reported measures might induce recall bias and social desirability bias. Disclosing IGD symptoms can be a sensitive issue for students during school-based surveys, although anonymity was guaranteed. Third, perceived short-term and long-term benefits and costs were each measured by a single item. While this approach follows precedent in time preference research [14] and minimizes participant burden in large school-based surveys, it has notable limitations. Single-item measures do not allow estimation of internal consistency reliability and may fail to capture the multidimensional nature of perceived benefits and costs (e.g., social, emotional, academic domains). Measurement error inherent in single items could attenuate observed relationships, suggesting our null findings should be interpreted cautiously. Future research should replicate our findings using validated multi-item scales that separately assess distinct domains of short-term and long-term benefits and costs. Fourth, our measures were limited in scope, and several important potential confounders were not assessed. We did not measure actual gaming time, which is a strong correlate of IGD severity and could independently influence perceived gaming outcomes. We also did not record game genre, despite evidence that different genres (e.g., MMORPGs vs. casual games) vary in their addictive potential and associated benefits/costs. Most critically, we did not assess depressive and anxiety symptoms, which are highly comorbid with adolescent IGD and could confound the relationships between cognitive perceptions and IGD symptoms (e.g., depression-related anhedonia may reduce perceived long-term benefits). Future studies should include these measures to isolate the unique contributions of perceived benefits and costs.

## Conclusion and implications

This study indicates the instability of IGD status and that higher levels of IGD symptoms predicted more perceived short- and long-term costs of gaming, while perceptions of costs or benefits did not significantly predict IGD symptoms. It enhances the understanding of the prospective associations between cognitive perceptions of internet gaming and adolescent IGD. These results offer preliminary evidence that may inform existing cognitive-behavioral models of behavioral addiction. The unidirectional relationship is consistent with the interpretation that experience-based belief updating, rather than cognitive distortions primarily driving symptom development. If replicated in future studies with stronger causal identification strategies, these findings support a differentiated, stage-sensitive framework for addressing IGD. The unidirectional relationship suggests that prevention and intervention must employ distinct strategies. In the prevention phase, prior to significant symptom escalation, efforts should target modifiable behavioral patterns and contextual factors. Effective strategies may include fostering balanced digital habits, developing rewarding offline interests, and educating families on constructive monitoring and communication, thereby building resilience before problematic use becomes entrenched. In the intervention phase, for individuals with established IGD, the clinically significant elevation in perceived costs provides a critical therapeutic entry point. Clinicians can systematically leverage these self-identified negatives, such as academic declines or social conflicts, as a catalyst for change. Within modalities like Motivational Interviewing [41], these cost perceptions can be validated and explored to resolve ambivalence, build intrinsic motivation, and bridge the gap toward behavioral commitment.

## Supporting information

S1 File Raw data of the studied variables related to perceived benefits and costs and internet gaming disorder. (DOCX)

S1 Data Dataset‌‌. (CSV)
