# Supplementary material for: Longitudinal associations between perceived benefits and costs of internet gaming and internet gaming disorder in adolescent gamers: A cross‑lagged structural equation model
Source: PLoS One. 2026 Jun 9;21(6):e0351550. doi: 10.1371/journal.pone.0351550 (PMC13249151; doi:10.1371/journal.pone.0351550)
Supplement: S1 File — (DOCX) [file pone.0351550.s001.docx]

Table A1. Associations between background variables and IGD symptoms at T1 by univariate linear regression models (N=1032).

|  | *β* | B | SE | 95%CI |
| --- | --- | --- | --- | --- |
| Age | -.01 | -.03 | .11 | (-.24, .19) |
| Sex (male ref. vs. female) | **-.13** | **-.57** | **.13** | **(-.82, -.31)** |
| Born in urban area (yes ref. vs. no) | .02 | .12 | .18 | (-.24, .48) |
| Living with both parents (no ref. vs. yes) | **-.12** | **-.71** | **.18** | **(-1.07, -0.35)** |
| Family socio-economic status (ref. ordinary or below) |  |  |  |  |
| Good | **-.12** | **-.49** | **.15** | **(-.77, -.20)** |
| Very good | -.05 | -.41 | .25 | (-.90, .08) |
| Refuse to answer/unknown | .03 | .29 | .29 | (-.27, .86) |
| Father's education level (ref. junior high school or below) |  |  |  |  |
| Senior high school to college | **-.10** | **-.42** | **.21** | **(-.84, -.003)** |
| Undergraduate or above | **-.17** | **-.74** | **.21** | **(-1.15, -.33)** |
| Refuse to answer/unknown | -.05 | -.74 | .52 | (-1.76, .27) |
| Mother's education level (ref. junior high school or below) |  |  |  |  |
| Senior high school to college | **-.24** | **-1.03** | **.20** | **(-1.42, -.64)** |
| Undergraduate or above | **-.25** | **-1.05** | **.19** | **(-1.44, -.67)** |
| Refuse to answer/unknown | -.05 | -.76 | .48 | (-1.69, .17) |

*Note.* β=Standardized coefficients; B=Unstandardized coefficients; SE=Standard error; ref.=Reference group; 95%CI=95% confidence intervals. Statistically significant background variables were highlighted in bold.

**Sensitivity Analysis**


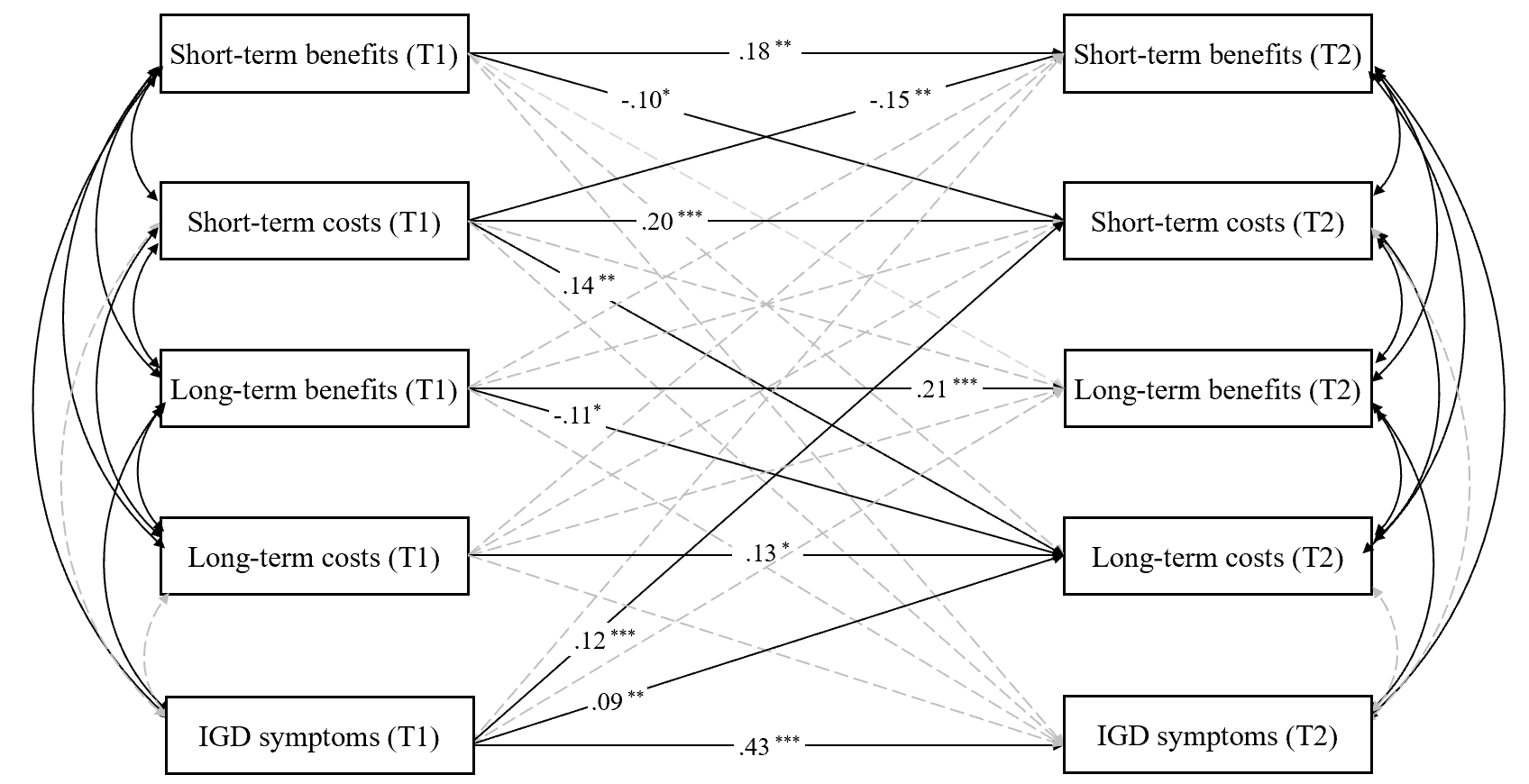


Fig. A1. Path analysis with perceived short-, long-term benefits, and perceived costs, and IGD symptoms. Model fit: Satorra-Bentler χ2/df=2.20, CFI=.97, TLI=.95, RMSEA=.03 (95%CI=.03-.04), SRMR=.02. Note. n.s.=Not significant. The standardized path coefficients are shown in the figure. The background variables (i.e., gender, family income, and parental education levels) were controlled for. * p< .05, ** p< .01, *** p< .001.


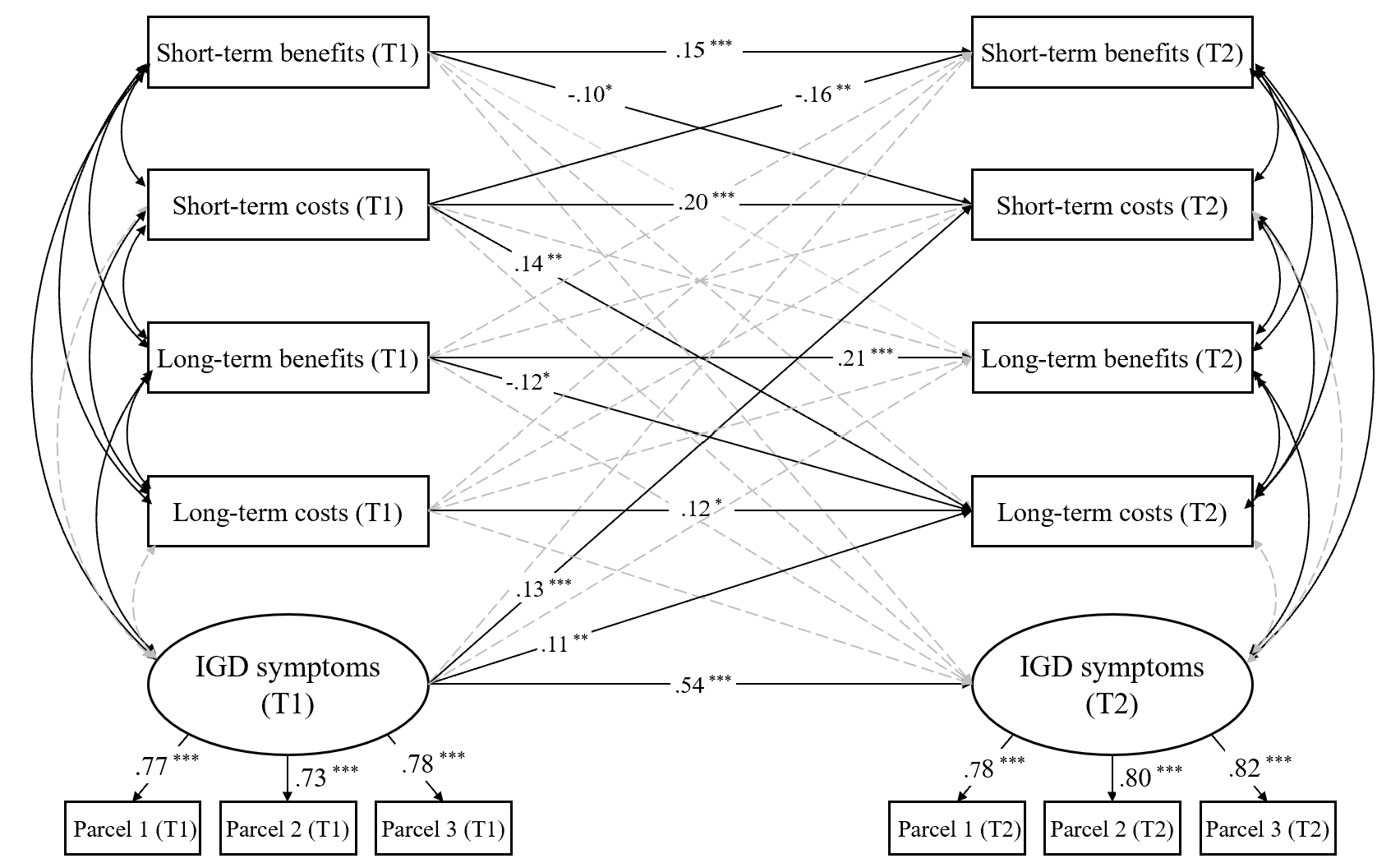


Fig. A2. Sensitivity analysis results using dataset imputed by mice in R. Model fit: Satorra-Bentler χ2/df=2.06, CFI=.98, TLI=.96, RMSEA=.03 (95%CI=.03-.04), SRMR=.03. Note. n.s.=Not significant. The standardized path coefficients are shown in the figure. The background variables (i.e., gender, family income, and parental education levels) were controlled for. * p< .05, ** p< .01, *** p< .001.
